# The biomechanical effects of clear aligner trimline designs and extensions on orthodontic tooth movement: a systematic review

**DOI:** 10.1186/s12903-024-05274-7

**Published:** 2024-12-20

**Authors:** Theerasak Nakornnoi, Watcharee Srirodjanakul, Rochaya Chintavalakorn, Peerapong Santiwong, Kawin Sipiyaruk

**Affiliations:** Department of Orthodontics, Faculty of Dentistry, Mahidol University, 6 Yothi Road, Ratchathewi, Bangkok, 10400 Thailand

**Keywords:** Biomechanical effects, Clear aligner, Orthodontics, Tooth movement, Aligner trimline

## Abstract

**Background:**

Clear aligner treatment (CAT) has emerged as an effective alternative to conventional multibracket systems in orthodontics. The trimline design and extension of aligners may significantly influence their biomechanical performance and tooth movement efficacy.

**Aim:**

To systematically review the biomechanical effects of different aligner trimline designs and extensions on orthodontic tooth movement.

**Methods:**

A systematic search was conducted across PubMed, Scopus, Embase, ProQuest Dissertations & Theses Global, and Google Scholar for studies published between January 2000 and August 2024. The review included any types of empirical research focusing on the influence of trimline of orthodontic aligners on tooth movement efficacy conducted between January 2000 and August 2024. The Risk of Bias In Non-randomized Studies of Interventions (ROBINS-I) tool was used for quality assessment.

**Results:**

Twelve studies met the inclusion criteria, all assessed as having low to moderate risk of bias. Aligner trimline design significantly influenced orthodontic tooth movement efficacy through two primary mechanisms: enhanced force delivery and increased aligner retention. Aligners with straight and extended margins generally exerted higher forces and moments compared to scalloped or shorter designs. This resulted in greater tooth displacement for certain movements, particularly intrusion, translation, tipping, and root torquing. Extended trimlines also demonstrated superior retention. However, the effects varied depending on the type of tooth movement.

**Conclusion:**

Aligner trimline designs and extensions can significantly influence biomechanical performance and tooth movement efficacy in CAT. Straight extended trimlines generally demonstrate superior force delivery and retention, leading to more predictable clinical outcomes. This could reduce the need for revisions, thereby decreasing overall treatment time and increasing patient satisfaction. However, further research is needed to investigate the interactions between aligner trimline designs and other factors to develop evidence-based guidelines for their optimal combination in various clinical scenarios.

## Introduction

Clear aligner treatment (CAT) has emerged as an effective alternative to conventional multibracket systems in orthodontics over past decades. Aligner therapy and digital treatment planning software had appeared to be increasingly important in current clinical practice [1]. This innovative approach offers superior aesthetics and comfort compared to traditional braces [2, 3]. Patients undergoing CAT report higher satisfaction with the treatment, particularly in eating and chewing categories [4]. In CAT, a virtual treatment setup of the desired tooth position and occlusion is meticulously performed by the clinician during the treatment planning stage. This setup has been shown to be clinically acceptable in terms of accuracy [5]. CAT utilizes a series of custom-made, removable aligners that incrementally move teeth towards predetermined positions through carefully planned force applications on specific areas of the dental crown [6]. The effectiveness of CAT in achieving precise tooth movements has made it a popular choice among patients and clinicians alike.

Recent research has focused on evaluating the efficacy of CAT in treating various malocclusions, including more severe cases [3, 7, 8]. While CAT has demonstrated effectiveness in certain orthodontic procedures such as leveling, aligning, and controlling intrusion and posterior buccolingual inclination, it faces challenges with more complex tooth movements. Scientific evidence has highlighted CAT’s limitations in controlling anterior tooth extrusion, rotating rounded teeth, managing anterior buccolingual inclination, and achieving bodily tooth movement [9]. The predictability of tooth movement in premolar extraction cases was shown to be undesirable with CAT [10]. However, another study demonstrated that CAT can achieve clinically acceptable outcomes comparable to those of conventional fixed appliances in controlling the buccolingual inclination of incisors in cases of mild to moderate malocclusions [3]. Moreover, a comparative analysis of treatment effectiveness and efficacy between clear aligners and fixed appliances, using the Peer Assessment Rating (PAR) index, revealed no significant difference in final scores between the two systems [11]. In addition, CAT has been proven to be effective in orthognathic surgical cases [12, 13]. These findings underscore both potential advantages and current limitations of CAT in orthodontic treatment, highlighting the need for further research to optimize its application across a broader range of orthodontic cases.

The efficacy of CAT is influenced by aligner design and manufacturing, including material composition, aligner thickness, trimline design, and the use of auxiliary devices. The mechanical performance of aligners is primarily dependent on the fabrication materials [14–17], with ongoing research focused on developing thermoplastic materials with enhanced mechanical, optical, and force delivery properties [18, 19]. The improvements in optical properties enhance the transparency and color stability of clear aligners, making them more aesthetically acceptable to patients [18]. Additionally, these advancements increase durability and enable the consistent application of orthodontic forces over extended periods, facilitating more precise and sustained tooth movement [20]. Aligner thickness, typically ranging from 0.50 to 1.50 mm, plays a crucial role in determining the appliance’s mechanical properties and, consequently, its effectiveness in tooth movement [17, 20]. Furthermore, the incorporation of aligner attachments significantly contributes to the precision of tooth movement, thereby optimizing the overall effectiveness of aligner therapy [21]. These various components are critical factors that must be carefully considered in the ongoing efforts to enhance the efficacy and versatility of CAT across a spectrum of orthodontic cases.

The morphology of the aligner trimline may influence the biomechanical performance of CAT. The gingival margin design of aligners, commonly known as the trimline, is an important determinant of both aligner retention and biomechanical force delivery [22–24]. These trimlines can be straight or scalloped, following the cervical margin of teeth, and may extend over varying areas of the attached gingiva. Recent research has identified the design of the aligner trimline as a potential factor influencing tooth movement efficacy [21, 25, 26]. However, a consensus has yet to be reached. This lack of a definitive conclusion, coupled with the significant impact of aligner trimline design on treatment outcomes, underscores the need for a comprehensive evaluation of existing evidence. Therefore, this systematic review aims to thoroughly examine the biomechanical effects of aligner trimline design and extension on tooth movement efficacy and retention across various types of orthodontic movements, providing clinicians with evidence-based guidance for optimizing aligner design in orthodontic practice.

## Materials and methods

### Review design

A systematic review methodology was adopted to comprehensively evaluate the effect of trimline design and extension on orthodontic aligner efficacy. This approach was chosen for its rigorous and standardized process in synthesizing evidence from multiple studies. The systematic review adheres to established scientific protocols for searching, screening, appraising, and synthesizing research findings [27]. This methodology would generate a thorough and objective evaluation of existing evidence on how aligner trimline designs could impact tooth movement outcomes.

### Search strategy

The PICO framework was utilized to structure our research question and guide the literature search. The central question, “Does clear aligner’s trimline design and extension affect orthodontic tooth movement?“, was broken down into Population (orthodontic patients using clear aligners), Intervention (clear aligners with specific trimline designs and extension variations), Comparison (clear aligners with standard trimline designs and extensions), and Outcome (tooth movement and retention). To enhance search sensitivity, the search terms derived from the PICO components combined the Intervention and Comparison under the single term ‘trimline’ without specifying variations or conventional designs (Table 1). This approach, which combines both components or excludes terms for ‘Comparison,’ has similarly been applied in systematic searches in previous review articles and appears to be an acceptable method for identifying relevant publications [28–31]. This structured approach facilitated a comprehensive and focused search across five databases: PubMed, Scopus, Embase, ProQuest Dissertations & Theses (PQDT) Global, and Google Scholar. Additionally, reference lists from the identified articles were thoroughly examined. The last search was conducted on 31 August 2024.

**Table 1:** Search terms developed according to PICO approach

*P* – Population	“orthodontic aligners”
I – Intervention	margin OR edge or trimline OR “trimming line”
C – Comparison	
O – Outcomes	“tooth movement” OR retention OR removability

### Inclusion and exclusion criteria

Any types of empirical research focusing on the impact of trimline design and extension of CAT on retention or tooth movement efficacy conducted between January 2000 and August 2024 were included in this review. Any of them which were not available in full-text were excluded. The inclusion and exclusion criteria were demonstrated in Table 2.

**Table 2:** Inclusion and exclusion criteria

Inclusion criteria	Exclusion criteria
- Empirical studies. - Studies evaluating effects of aligner’s trimline designs or extensions on tooth movement or retention. - Studies published between January 2000 and August 2024.	- Studies not relevant to retention or tooth movement generated by aligners. - Studies not available in full-text.

### Article selection

Following the systematic search, two researchers (T.N. and W.S.) independently screened the titles, abstracts, and full-texts based on the established inclusion and exclusion criteria. Any disagreements in article selection between the researchers were addressed through discussion and consultation with a third researcher (K.S.).

### Risk of bias assessment

A risk of bias assessment of articles included in this systematic review was performed independently by two researchers (T.N. and W.S.). Any disagreements on the risk of bias assessment were resolved by discussing with the third researcher (K.S.).

To evaluate the quality of the included non-randomized studies, this systematic review employed the Cochrane Collaboration’s ‘Risk of Bias In Non-randomized Studies of Interventions (ROBINS-I)’ tool [32]. This tool assesses bias across seven domains, providing a comprehensive evaluation of each study’s methodological rigor. The assessment categories range from low to critical risk of bias, allowing for a nuanced understanding of each study’s strengths and limitations. By systematically applying this tool, the synthesis could ensure a transparent and standardized approach to evaluating the reliability of findings.

### Data extraction and synthesis

A structured data extraction process was implemented to systematically collect relevant information from each included study. Eight key categories were identified for data extraction: study design, research objectives, margin variations, tooth studied, outcome measurement, results, conclusion, and risk of bias assessment. This comprehensive approach ensures that all pertinent information is captured for analysis (Table 3). The extracted data were then synthesized narratively, allowing for a detailed and contextualized interpretation of the findings across all included studies.

**Table 3:** Data extraction of included studies

	Authors (Year)	Trimline variation	Outcome assessment	Tooth studied	Objectives	Study design	Key findings	Risk of bias
1	**Lyu et al. (2022)** [33]	1. Scalloped − 2 mm 2. Scalloped 0 mm 3. Scalloped 2 mm 4. Straight − 2 mm 5. Straight 0 mm 6. Straight 2 mm	Tooth movement: En-masse retraction	Whole maxillary arch	To assess the effects of gingival margin design on force expression and stress distribution during upper en-masse retraction in extraction treatment.	Finite element study	Aligners with a margin height of -2 mm showed significantly lower stress, particularly with scalloped cuts. Straight margin aligners generated higher stress than scalloped ones, but the difference was less pronounced at a 2 mm height.	Moderate
2	**Elshazly et al. (2023)** [34]	1. Scalloped 0 mm 2. Scalloped 2 mm 3. Straight 0 mm 4. Straight 2 mm	Tooth movement: Palatal translation	Tooth 11	To assess the effects of gingival margin designs on forces delivered by orthodontic aligners during the bodily movement of the maxillary central incisor.	Finite element study	Aligners with straight margins exerted higher forces than those with scalloped margins, with force increasing as margin extension increased.	Moderate
3	**Elshazly et al. (2024)** [23]	1. Scalloped 0 mm 2. Scalloped 2 mm 3. Straight 0 mm 4. Straight 2 mm	Tooth movement: Palatal translation	Tooth 11	To investigate the impact of gingival margin designs on the biomechanical behavior of orthodontic aligners.	Finite element study	Aligners with straight extended margins positively impact force distribution and control of tooth movement.	Moderate
4	**Elshazly et al. (2024)** [35]	1. Straight 0 mm 2. Straight 2 mm	Tooth movement: Facial translation, distalization, and extrusion	Tooth 11	To investigate the impact of trimming line designs on the biomechanical performance of orthodontic aligners.	Finite element study	Aligners with straight extended margins provide better control of tooth movement and can serve as an alternative to attachments in some cases.	Moderate
5	**Karsli et al. (2024)** [36]	1. Straight 0.5 mm 2. Straight 2 mm	Tooth movement: Arch expansion	Maxillary first and second molars	To evaluate the effects of different trimline extensions on maxillary first and second molars during arch expansion.	Finite element study	Aligners with high trimline reduced buccal tipping of maxillary molars during arch expansion.	Moderate
6	**Karsli et al. (2024)** [37]	1. Straight 0.5 mm 2. Straight 2 mm	Tooth movement: Utilization of Class II inter-maxillary elastic	Mandibular anterior teeth and molars	To evaluate the effect of trimline extension of orthodontic aligners when combined with Class II intermaxillary elastics.	Finite element study	Aligners with high trimlines effectively controlled mandibular incisor proclination and mesial tipping of mandibular molars during clear aligner treatment with Class II elastics.	Moderate
7	**Gao et al. (2017)** [38]	1. Scalloped 0–1 mm 2. Straight 3–4 mm 3. Straight 6–7 mm	Tooth movement: Palatal tipping and intrusion	Tooth 11	To assess the effects of gingival margin height on the force system from orthodontic aligners during tipping and intrusion of the maxillary central incisor.	Force and moment sensors	Aligners with longer margins (3–4 mm and 6–7 mm) deliver significantly greater intrusion force and tipping moment than edgeless aligners. No significant difference in force or moment was observed between the 3–4 mm and 6–7 mm aligners.	Moderate
8	**Brown et al. (2021)** [39]	1. Scalloped 0 mm 2. Straight 0.75 mm 3. Straight 1.5 mm	Tooth movement: Palatal root movement	Tooth 21	To assess the effects of gingival margin design on strain distribution and force system from orthodontic aligners during labial tipping of the maxillary central incisor.	Force measuring device, and Digital Image Correlation (DIC)	Bucco-lingual force and root torquing moment from aligners vary significantly by margin design, with greater differences between straight and scalloped designs than among straight cut heights. The impact of varying gingival margins on force output is 20–50%.	Low
9	**Elshazly et al. (2022)** [24]	1. Scalloped 0 mm 2. Scalloped 2 mm 3. Straight 0 mm 4. Straight 2 mm	Tooth movement: Palatal translation	Tooth 11	To assess the effects of gingival margin designs on stress distribution and forces from orthodontic aligners during the bodily movement of the maxillary central incisor.	Pressure-sensitive film	Aligners with straight extended margins exhibited the highest active force, active pressure, and passive pressure, delivering more uniform force transfer and stress distribution compared to scalloped margins. Significant differences in force and pressure were particularly noted at the cervical area of the tooth.	Moderate
10	**Traversa et al. (2024)** [40]	1. Straight 0 mm 2. Straight 2 mm	Tooth movement: Palatal translation, mesial translation, intrusion	Tooth 11, 13, 16, and neighboring teeth	To report the biomechanical performance of orthodontic aligners with varying trimline heights during three types of translational tooth movements.	Orthodontic force simulator	Aligners with high trimline enhances control over orthodontic movements.	Low
11	**Cowley et al. (2012)** [22]	1. Scalloped 0 mm 2. Scalloped 2 mm 3. Straight 0 mm 4. Straight 2 mm	Retention	Whole maxillary arch	To evaluate the impact of gingival margin design on orthodontic aligner retention.	Laboratory setting: Retentive pull-off test	Aligners with 2 mm straight margins showed significantly higher retention than scalloped margins at the same height, while 0 mm aligners exhibited no significant difference between straight and scalloped margins.	Low
12	**Takara et al. (2022)** [41]	1. Straight at HOC 2. Straight 0 mm 3. Straight 2 mm	Retention	Whole maxillary arch	To assess the impact of gingival margin height on orthodontic aligner retention.	Laboratory setting: Specific measuring device	Aligners with longer margins show significantly higher retention.	Low

## Results

### Articles identified from the search

**Fig. 1:**
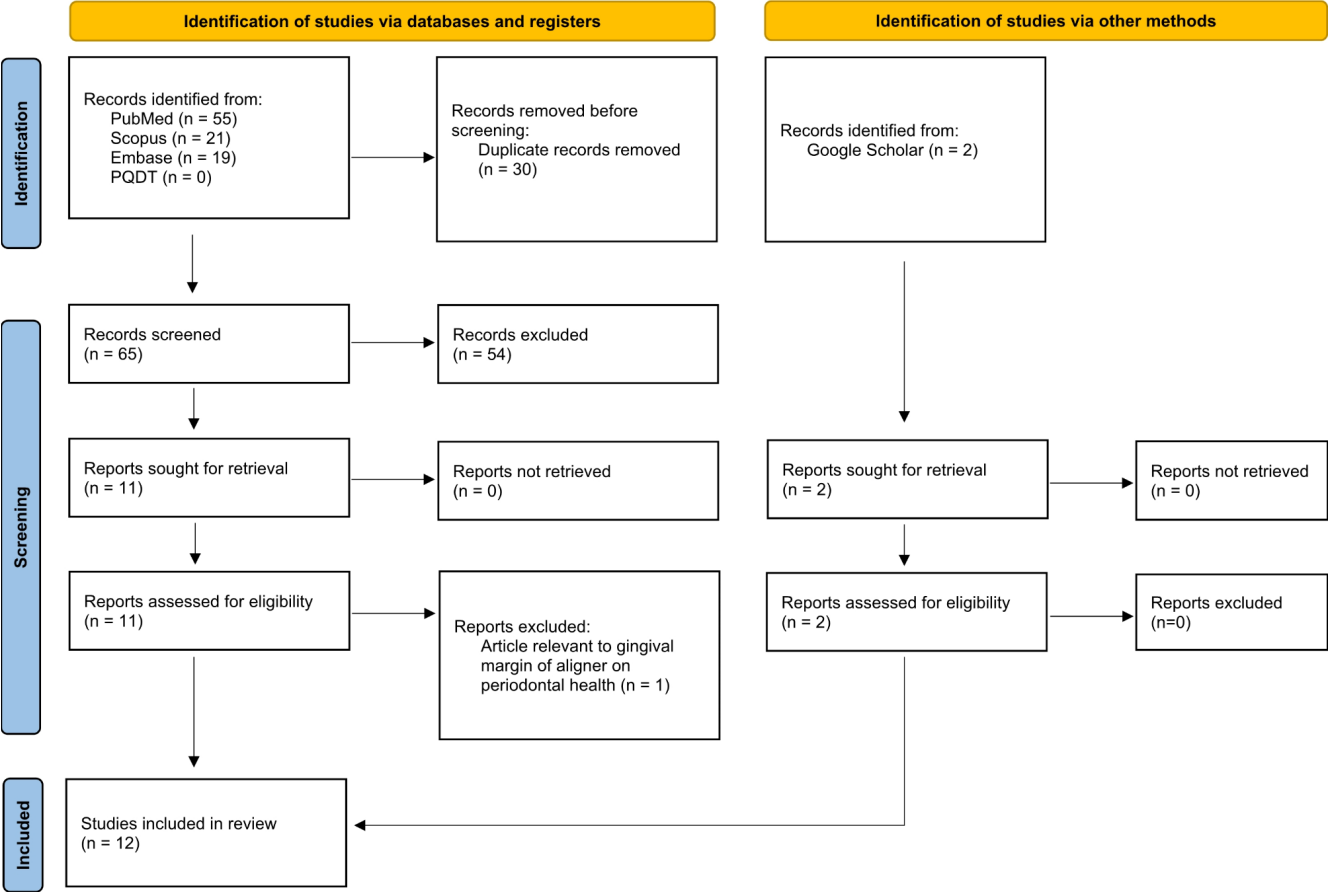
PRISMA 2020 flow diagram for new systematic reviews which included searches of databases, registers and other sources

The study selection process is illustrated in the PRISMA flow diagram (Fig. 1). The electronic database search yielded 95 articles (PubMed: 55, Scopus: 21, Embase: 19, PQDT: 0). After removing 30 duplicates, 65 articles were screened by title and abstract. Based on the inclusion and exclusion criteria, 54 articles were excluded at this stage. Two additional articles were identified through Google Scholar, resulting in 13 full-text articles assessed for eligibility. One article was excluded as it focused on the effects of aligner gingival margin on periodontal health rather than tooth movement. Finally, 12 articles were included in this systematic review.

### Characteristics of articles included

Of twelve included articles, six studies utilized Finite Element Method (FEM), with three focusing on single tooth movements of the upper maxillary central incisor [23, 34, 35] and three examining multiple-tooth movements, including en-masse retraction of upper anterior teeth [33], upper arch expansion [36], and utilization of Class II elastic traction [37]. The remaining six studies employed laboratory techniques, in which three used force measuring devices/sensors on single teeth [38, 39] and adjacent teeth [33], two conducted retentive tests on upper dental models [22, 41], and one utilized pressure-sensitive films to visualize force, pressure, and stress distribution on the dental crown surface during palatal translation of the upper central incisor [24].

The efficacy of tooth movement was evaluated through various parameters across these studies. Seven studies focused on force and moment delivery from aligner to tooth [23, 24, 34, 35, 38–40], while tooth displacement was evaluated in five studies [23, 33, 35–37]. Stress distribution in the periodontal ligament (PDL) was measured in four studies [33, 35–37], and stress on the dental crown was investigated in two studies [24, 33]. Additionally, one study examined strain [39], and two studies focused on aligner retention, an important factor influencing the efficacy of tooth movement [22, 41]. This diverse range of methodologies and parameters provides a comprehensive overview of the current research on CAT efficacy.

### Risk of bias assessment

All included studies were assessed as having low to moderate risk of bias. FEM studies were consistently graded as moderate risk due to possible bias in the selection of participants (dentition models) into the study. Most laboratory studies were assessed as having low risk of bias, with one article showing moderate risk in the measurement of outcomes [38]. This diverse range of methodologies and parameters (Table 4), along with the risk of bias assessment, provides a comprehensive overview of the current research on CAT efficacy.

**Table 4:** Methodological limitations of included studies

	Methodological limitations	Authors (Year)
**Finite element studies**	- Potential bias in selection of models into analysis.	Lyu et al. (2022) [33] Elshazly et al. (2023) [34] Elshazly et al. (2024) [23] Elshazly et al. (2024) [35] Karsli et al. (2024) [36] Karsli et al. (2024) [37]
	- Lack of the simulations involving PDL and/or other teeth.	Elshazly et al. (2023) [34] Elshazly et al. (2024) [23] Elshazly et al. (2024) [35]
**Laboratory studies**	- Potential wear of models during testing. Forces from repeated aligner use can degrade surface integrity, affecting fit and force application and transmission. - Lack of PDL and saliva in settings.	Cowley et al. (2012) [22] Gao et al. (2017) [38] Brown et al. (2021) [39] Elshazly et al. (2022) [24] Takara et al. (2022) [41] Traversa et al. (2024) [40]

### Effects of aligner trimline designs and extensions on tooth movement

The efficacy of orthodontic tooth movement by aligners can be measured directly through tooth displacement or indirectly through force and moment delivered from the aligner, stress distribution in PDL, or strain and stress on the dental crown.

#### Tooth displacement

Five FEM studies measured tooth displacement in relation to aligners with different trimlines. Results varied among studies. For en-masse retraction, aligners with straight margins showed significantly greater tooth movement and control (expressed by a larger root-to-crown movement ratio) compared to those with scalloped margins [33]. However, no significant differences were observed in initial displacement for palatal translation, facial translation, distalization, and extrusion of upper central incisors among different margin designs and extensions [23, 35]. Aligners with low trimlines demonstrated larger tooth displacement than those with high trimlines in both arch expansion and Class II elastic traction [36, 37].

#### Force and moment delivery

Seven articles measured force and moment delivery from aligners with various trimline designs and extensions, revealing similar trends regardless of the methods used across the studies. Aligners with straight and extended margins generally delivered the highest intrusive and translational forces, tipping, and root torquing moments, while scalloped and short margins exerted the lowest forces and moments [23, 24, 34, 35, 38–40]. Among these, two laboratory studies further highlighted these differences. Aligners with a 3–4 mm edge width delivered higher intrusive force and a greater palatal tipping moment than those with a 0–1 mm edge width, while a 6–7 mm edge width showed no significant difference from 3 to 4 mm [38]. Additionally, the difference in force and moment for palatal root movement was more pronounced between straight and scalloped margins than between different extensions of straight margins [39].

#### Stress distribution in PDL

Four FEM studies reported PDL stress distribution, showing varied results of a pattern of compressive and tensile stress concentration. In the case of en-masse retraction, straight trimlines exhibited higher stress than scalloped designs, but the impact was minimal when the margin was extended above the gingival zenith [33]. No noticeable differences were observed among different aligner designs during translation and extrusion [35]. Higher PDL stress distribution was identified in aligners with low trimlines compared to high trimlines in both arch expansion and Class II elastic traction [36, 37].

#### Strain and stress on dental crown

One laboratory study and two FEM articles reported similar pattern of strain and stress exerted by aligner on dental crown. During translation, root movement, and anterior retraction, strain and stress on the dental crown were significantly higher in aligners with straight extended margins, with more pronounced differences in cervical areas on the tooth surface [24, 33, 39].

### Effects of aligner trimline designs and extensions on retention

Aligner retention impacts the efficiency of orthodontic tooth movement. Two laboratory studies evaluated retention of aligners with different gingival margin designs and extensions by measuring retentive force during aligner removal [22, 41]. They concluded that aligners with extended trimlines were more retentive than shorter ones with similar designs, and straight trimlines were more retentive than scalloped margins.

## Discussion

This systematic review pointed out the increasing interest in the impact of aligner trimline on orthodontic tooth movement in recent years, with ten out of twelve included studies published from 2021 to 2024 [23, 24, 33–37, 39–41]. This recent surge in research activity likely reflects the growing popularity of clear aligner therapy and the recognized need for evidence-based optimization of aligner design. However, it underscores that this emerging field currently lacks well-established clinical trials to validate the findings from these predominantly in vitro and simulation-based studies. The included studies were classified according to method of evaluation into two groups: half utilized laboratory settings, while the other half implemented FEM for result evaluation. FEM is widely adopted by orthodontic field providing data on physiologic reactions in tissues through visualization of areas of stress created from orthodontic force application [42]. This division highlights the diverse approaches used to investigate aligner gingival margin effects, balancing tangible, real-world data from laboratory studies with the complex biomechanical modeling capabilities of FEM simulations.

The straight extended trimline in CAT has been shown to be biomechanically effective, particularly in terms of force and moment transfer across various types of tooth movement [23, 24, 34, 35, 38–40], and in exerting significantly higher stress on dental crowns, especially at cervical areas [24, 39]. This design aligns well with the fundamental principle that orthodontic tooth movement relies on the relationship between the applied force vector and the tooth’s center of resistance (CR) [43, 44]. The force application areas closer to the CR, which are found in aligners with straight extended trimlines, are necessary for complex tooth movements such as translation and root movement, supporting the notion that modifying aligner geometries can improve root control [45]. The benefits of extended trimline designs extend beyond simple tooth movements. In en-masse retraction, this design demonstrates superior force delivery and control [33]. For maxillary arch expansion, aligners with high trimlines result in less undesirable buccal tipping of molars compared to those with low trimlines [36]. Similarly, during Class II elastic traction, high-trimline aligners show reduced mandibular incisor proclination and mesial tipping of mandibular molars [37]. These findings collectively support the biomechanical advantage of straight extended trimlines in CAT.

The PDL stress distribution, characterized by compressive and tensile stress concentrations under orthodontic loading, has been found to be consistent with tooth displacement findings [23, 33, 36, 37]. This stress magnitude within the periodontium acts as the primary mediator of tooth movement [46]. Orthodontic forces induce tooth movement by generating either tensile or compressive stress in the periodontium, which contributes to alveolar bone remodeling through the recruitment of osteoblasts and osteoclasts [47, 48]. While previous studies have established that the intensity of tensile and compressive stress induced by a thermoplastic appliance is related to appliance thickness [44, 49], this review highlights that trimline design and extension also play crucial roles in PDL stress distribution, expanding our understanding of the biomechanical factors influencing CAT.

Aligners with straight and extended trimlines provide higher retention compared to those with short and scalloped trimline designs [22, 41], thereby increasing the ability to achieve better control over orthodontic tooth movement. However, this design consideration requires a nuanced approach in clinical practice. For patients with pre-existing retentive conditions such as gingival recession, cervical abfraction, black triangles, and severe dental proclination [40], the potential for discomfort during aligner insertion and removal should be carefully evaluated. Moreover, in cases where gentler force application is necessary, extended trimlines may not be suitable due to the risk of overloading periodontal structures [39]. In such cases, scalloped trimlines might offer a more appropriate alternative. However, the impact of trimline design on periodontal health presents an interesting dichotomy. The protective effect of extended trimlines, demonstrating that aligners with extended margins helped prevent plaque deposition and mechanical irritation compared to edgeless aligners, which worsened periodontal health by facilitating plaque buildup and causing mechanical irritation during removal [50]. Conversely, there are concerns that the gingival coverage of extended trimlines may be potentially less hygienic and requires more detailed impressions or scans of the surrounding gingiva [51]. Given these conflicting considerations, the biomechanical advantages of extended trimline designs and extensions should be carefully weighed against patient-specific factors to ensure optimal treatment outcomes while minimizing potential adverse effects.

In addition to aligner trimline designs and extensions, other modifiable factors have been found to influence tooth movement outcomes. These factors include manufacturing materials and the presence of auxiliaries such as attachments, elastics, and miniscrews [21]. A network meta-analysis demonstrated that the materials used in fabricating clear aligners significantly affected tooth movement outcomes [52]. The presence of attachments has been shown to enhance the effectiveness of tooth movement, particularly for bodily tooth movement, root torque, and rotation [53–55]. Interestingly, no significant differences were observed among various shapes and sizes of attachments when considering the same type of aligner material [15, 56]. By integrating these factors, orthodontists may be able to optimize treatment outcomes and potentially improve the predictability of tooth movement with clear aligner therapy.

While this systematic review demonstrates the significant influence of aligner trimline designs on tooth movement outcomes, several research limitations need to be discussed. The heterogeneity in study methodologies precluded a meta-analysis and made direct comparisons between studies challenging. These limitations underscore the need for standardized research protocols in future investigations. Additionally, most research has focused on individual factors in isolation, potentially overlooking synergistic or antagonistic effects when multiple variables are combined. Future research should investigate the biomechanical principles underlying the interactions between these variables, potentially through finite element analysis or in vitro simulations. Furthermore, longitudinal clinical trials comparing different combinations of these factors could provide more robust evidence for their relative efficacy. Further investigation of these areas will contribute to a better understanding of clear aligner efficacy, potentially enhancing treatment predictability and efficiency in clinical practice.

## Conclusions

This systematic review provides compelling evidence that the gingival margin design and extension of clear aligners play a crucial role in determining their biomechanical performance and efficacy in orthodontic tooth movement. Orthodontic aligners with straight and extended trimlines generally exhibit superior force and moment delivery, particularly for intrusion, translation, tipping, and root torquing movements. These designs also offer enhanced retention. However, the interactions between trimline design and other factors, such as aligner material properties and the use of attachments, require further investigation. Future research should focus on clinical trials to validate these findings in diverse patient populations and explore the synergistic effects of various aligner design elements.

## Data Availability

The data that support the findings of this study are available from the corresponding author, up-on reasonable request.
